# Intrinsic Connectivity Changes Mediate the Beneficial Effect of Cardiovascular Exercise on Sustained Visual Attention

**DOI:** 10.1093/texcom/tgaa075

**Published:** 2020-10-09

**Authors:** Nico Lehmann, Arno Villringer, Marco Taubert

**Affiliations:** Department of Neurology, Max Planck Institute for Human Cognitive and Brain Sciences, Leipzig 04103, Germany; Department of Sport Science, Faculty of Human Sciences, Institute III, Otto von Guericke University, Magdeburg 39104, Germany; Department of Neurology, Max Planck Institute for Human Cognitive and Brain Sciences, Leipzig 04103, Germany; Mind and Brain Institute, Charité and Humboldt University, Berlin 10117, Germany; Department of Sport Science, Faculty of Human Sciences, Institute III, Otto von Guericke University, Magdeburg 39104, Germany; Center for Behavioral and Brain Science (CBBS), Otto von Guericke University, Magdeburg 39106, Germany

**Keywords:** attention, cardiovascular exercise, cognition, MRI, neuroplasticity

## Abstract

Cardiovascular exercise (CE) is an evidence-based healthy lifestyle strategy. Yet, little is known about its effects on brain and cognition in young adults. Furthermore, evidence supporting a causal path linking CE to human cognitive performance via neuroplasticity is currently lacking. To understand the brain networks that mediate the CE–cognition relationship, we conducted a longitudinal, controlled trial with healthy human participants to compare the effects of a 2–week CE intervention against a non-CE control group on cognitive performance. Concomitantly, we used structural and functional magnetic resonance imaging to investigate the neural mechanisms mediating between CE and cognition. On the behavioral level, we found that CE improved sustained attention, but not processing speed or short-term memory. Using graph theoretical measures and statistical mediation analysis, we found that a localized increase in eigenvector centrality in the left middle frontal gyrus, probably reflecting changes within an attention-related network, conveyed the effect of CE on cognition. Finally, we found CE-induced changes in white matter microstructure that correlated with intrinsic connectivity changes (intermodal correlation). These results suggest that CE is a promising intervention strategy to improve sustained attention via brain plasticity in young, healthy adults.

## Introduction

Cognitive functions can be viewed as the behavioral outcome of collaborative processing of sensory information by distributed but interconnected neural systems (Mesulam 1998). Based on psychometric theory and supported by neuroscientific evidence of specialized neural networks (Eich et al. 2020), cognitive functions can be subdivided into domains such as processing speed, reasoning, and memory, to name but a few (McGrew 2009). Importantly, an increasing number of studies have found that the properties of functional and supporting anatomical networks possess predictive validity, with respect to neurocognitive abilities (Gabrieli et al. 2015; Tavor et al. 2016), therefore providing a relevant biological target for neuro-enhancement strategies.

Against this background, it is not surprising that recent years have witnessed an increased interest in promoting cognitive functioning with interventions designed to influence the brain (Dresler et al. 2013; Clark and Parasuraman 2014). In this regard, cardiovascular exercise (CE) is considered a promising intervention strategy for healthy and diseased human populations of all ages, being low in cost, low in risk, and easily applicable (Erickson et al. 2019). This view is supported, on the one hand, by recent meta-analytic evidence demonstrating that CE is robustly related to improved cognitive functioning on the behavioral level (Smith et al. 2010; Roig et al. 2013; Ludyga et al. 2020). On the other hand, CE has also been associated with several cognition-related brain biomarkers, among them synaptic, glial, vascular, and myelin plasticity or the expression of plasticity-related genes and growth factors (Voss, Vivar, et al. 2013). In humans, a commonly used method to study CE-induced plasticity is magnetic resonance imaging (MRI) (Stillman et al. 2016; van der Stouwe et al. 2018). Use of MRI is based on the assumption that the bulk effect of the aforementioned cellular- and molecular-level events is at least partially captured by the imaging signal (Logothetis et al. 2001; Blumenfeld-Katzir et al. 2011; Sampaio-Baptista et al. 2013; Keifer et al. 2015).

On closer inspection, several important gaps in the field of CE-induced cognitive enhancement must be noted. It is especially apparent that previous research has mainly focused on the age groups at the extremes of the lifespan. Childhood and adolescence (≤ 18y) on the one end of the spectrum and older and senescent adults (>50 years) on the other (Northey et al. 2018; Erickson et al. 2019; Singh et al. 2019; Valkenborghs et al. 2019). Although the first long-term intervention studies among young and middle-age healthy adults indicate that CE can improve neurocognitive performance in domains like memory (Stroth et al. 2009; Hötting et al. 2012) and executive control (Stroth et al. 2010; Johann et al. 2016; Eather et al. 2019; Stern et al. 2019; Mekari et al. 2020), the available data for this age range are insufficient enough that a grade of evidence is “not assignable” (Erickson et al. 2019; see also Ludyga et al. 2020).

Similarly and independent of age group, the tacit assumption that CE-induced cognitive enhancement is a result of plasticity of task-relevant brain networks (Voss, Vivar, et al. 2013) has not yet undergone rigorous testing (cf., Stillman et al. 2016). Although it is widely accepted that cognitive functions require concerted activity of interconnected cortical and subcortical brain regions (Mesulam 1998; Bullmore and Sporns 2009), previous CE studies using MRI have focused predominantly on the hippocampal memory system (van der Stouwe et al. 2018). Interestingly, recent long-term intervention studies conducted with different age groups have reported evidence for other putative transfer mechanisms of CE, among them changes in prefrontal cortical thickness (Stern et al. 2019), structural (Voss, Heo, et al. 2013; Mueller et al. 2015; Svatkova et al. 2015), and functional connectivity (Voss et al. 2010; McGregor et al. 2018; Prehn et al. 2019) as well as cerebral perfusion (Maass et al. 2015). Unfortunately, besides one notable exception (Maass et al. 2015), these studies did not address whether brain changes mediate the association between CE and changes in cognition (Stillman et al. 2016).

In sum, our knowledge of the effects of CE on neurocognitive functions in young adults is based on very limited data. This lack of evidence might be rooted in the fact that this age group is considered the least vulnerable in terms of mental health and cognition. However, this must not obscure the fact that several, CE-modifiable (Pedersen and Saltin 2015), risk factors for subjective or actual cognitive impairment are already prevalent in young age, among them overweight (Smith et al. 2011) or elevated blood pressure (Joosten et al. 2013; Schaare et al. 2019). Furthermore, extensive research in psychology supports the idea that general cognitive ability is an important predictor of job performance (Schmidt and Hunter 1998) and academic achievement (Richardson et al. 2012; Ruffing et al. 2015). This further reinforces that a better understanding of the influence of CE on cognition and of the underlying neural transfer mechanisms in young adults is warranted.

To address these issues, we used a controlled study design with longitudinal cognitive testing and multimodal MRI. Here, we focus on blood-oxygen-level-dependent (BOLD) signal fluctuations in the resting-state (Raichle et al. 2001) and diffusion-weighted MRI (Pierpaoli and Basser 1996), allowing us to study intrinsic functional connectivity and microstructural features of white matter, respectively. Analyzing both structural and functional networks is reasonable against the background that structural and functional connectivity are intricately linked to each other (Bullmore and Sporns 2009; Greicius et al. 2009; Honey et al. 2009; Webb et al. 2020), such that a profound understanding of CE’s influence on the brain benefits from an integrated view on both aspects of connectivity. Three cognitive tests predominantly objectifying sustained attention, information processing speed, and short-term memory were chosen as behavioral endpoints because they were seldom considered in previous studies with young, healthy adults (see above), although these cognitive domains have shown to be susceptible to influence by CE (Smith et al. 2010; Ishihara et al. 2020).

There is an ongoing debate surrounding the question of “optimal” exercise regimens (e.g., in terms of exercise type, duration of the intervention and individual training sessions, intensity, frequency) with respect to brain plasticity and cognitive functions (van Praag et al. 2014; Erickson et al. 2019). Evidence from animal studies suggests that CE of short duration (≤ 2 weeks) is sufficient to trigger changes in plasticity-related genes, neurotrophins, and neuronal and non-neuronal tissue (Neeper et al. 1995; Fabel et al. 2009; Gómez-Pinilla et al. 2011; Simon et al. 2011; Abel and Rissman 2013; Bechara et al. 2014; Brockett et al. 2015; Sølvsten et al. 2018; Cefis et al. 2019). Importantly, MRI has shown to be sensitive to detect neuroplastic changes in response to brief learning (Blumenfeld-Katzir et al. 2011; Sagi et al. 2012; Taubert et al. 2016) as well as CE interventions (Sumiyoshi et al. 2014). Regarding exercise intensity, the existing evidence suggests that CE training that predominantly strains the anaerobic-lactic energy system leads to an augmented response of plasticity-related neurotrophins (Afzalpour et al. 2015; Saucedo Marquez et al. 2015). Furthermore, high-intensity exercise induces a beneficial pattern of shear stress acting on the cerebral vasculature, which in turn induces cerebral remodeling and the production and secretion of neurotrophins (Prigent-Tessier et al. 2013; Calverley et al. 2020; Carr and Ainslie 2020). For these reasons, we opted for a comparably short CE intervention (2 weeks) specifically designed to repeatedly strain the anaerobic-lactic energy system (Brooks 2018; Hashimoto et al. 2018; Magistretti and Allaman 2018). We hypothesize that this type of CE enhances cognition in young adults via affecting task-relevant structural and functional brain properties.

## Materials and Methods

### Participants and Experimental Design

A total of 50 healthy, right-handed adults aged 18–35 years (2 dropouts due to illness or injury) were assigned to a CE intervention or control group (see Table 1 for group characteristics). All subjects had a comparable level of education (A-levels). A subset of 34 participants was part of a previously published randomized controlled trial on the effects of CE on subsequent motor learning (Lehmann et al. 2020). These data were collapsed with another 16 participants who were not randomly assigned to the experimental conditions, but who underwent the same procedures as the intervention group in Lehmann et al. (2020). Note that all MRI measurements were conducted on the same 3 T system (no updates during the study period). Exclusion criteria were contraindications to MRI, body mass index (BMI) > 30 kg/cm^2^, a history of neuropsychiatric diseases, left-handedness, self-reported physical activity of >4 h/week, and past or present performance-oriented participation in endurance and/or coordinative-demanding sports. The study was carried out in accordance with the Declaration of Helsinki and approved by the Ethics Committee of the University of Leipzig. Subjects gave their informed written consent and underwent neurological examination as assessed by a credentialed physician prior to participation.

**Table 1:** Between-group comparison of demographic, anthropometric, and aerobic fitness data at baseline

	Exercise (*n* = 32)	Control (*n* = 16)	Exercise vs. control
Age (years)	25 (6)	23.5 (4)	*U*(16, 32) = 189.5, *P* = 0.50
Sex (♂/♀)	12/20	5/11	χ^2^(1) = 0.18, *P* = 0.67
Height (m)	1.74 (0.09)	1.74 (0.09)	*t*(32.7) = 0.05, *P* = 0.96
Weight (kg)	67.88 (10.68)	66.5 (9.56)	*t*(31.87) = −0.62, *P* = 0.54
BMI (kg/m^2^)	22.29 (2.12)	21.85 (2.08)	*t*(30.71) = −0.69, *P* = 0.50
Handedness (Oldfield 1971)	86 (21)	100 (20)	*U*(16, 32) = 295, *P* = 0.27
IAT (W/kg)	2.00 (0.46)	1.88 (0.27)	*U*(16, 32) = 232, *P* = 0.61
P_3_ (W/kg)	2.02 (0.41)	1.85 (0.48)	*t*(26.35) = −1.21, *P* = 0.24

Note: Descriptive statistics refer to frequencies (in the case of χ^2^ test), median, and interquartile range (in case of Mann–Whitney *U* test), or mean and SD (in case of Welch’s test). IAT, individual anaerobic threshold determined with the method proposed by Dickhuth et al. (1999); P_3_, workload at a fixed lactate concentration of 3 mmol/L.

**Figure 1:**
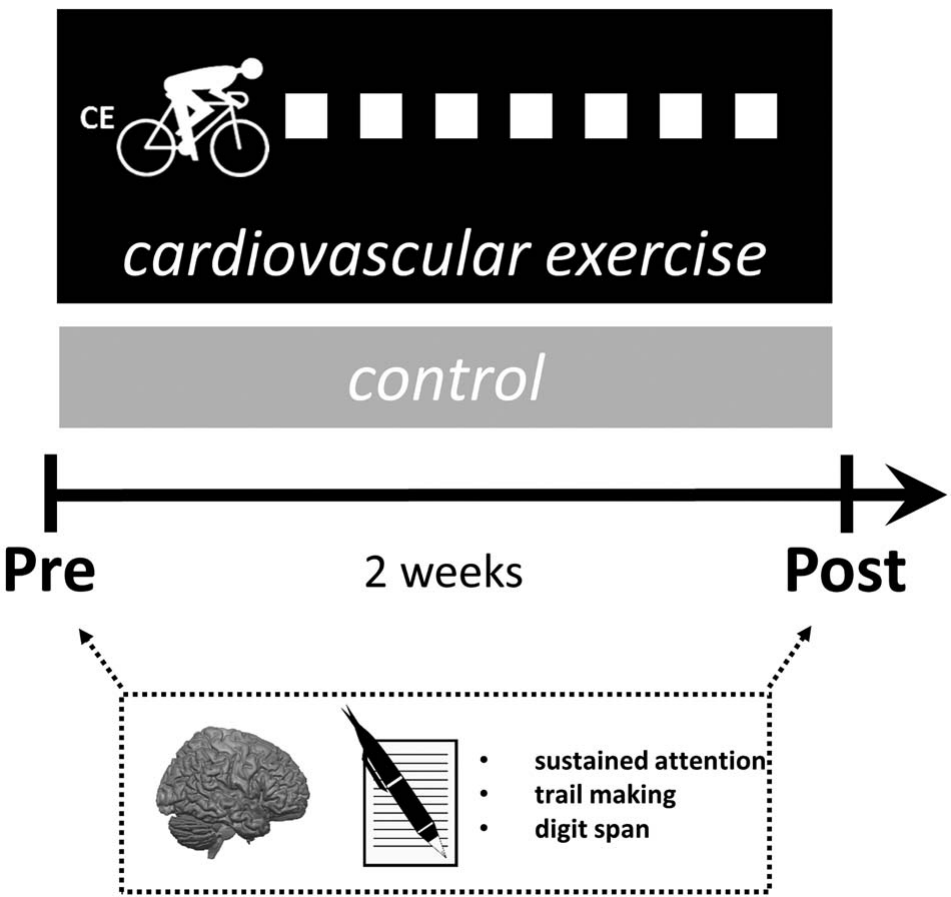
Overview of the experimental design. Subjects were assigned to either 2 weeks of CE or no exercise (life as usual). White squares depict 7 training sessions subjects in the CE group engaged in. MRI measurements to assess exercise-related neuroplasticity and cognitive assessments (paper-and-pencil tests) were conducted before (Pre) and after the intervention (Post). Furthermore, all subjects underwent a cardiovascular fitness assessment to determine aerobic fitness levels and to derive intensity prescriptions for the intervention group’s training schedule.

Cognitive assessments (paper-and-pencil tests) and MRI measurements were undertaken before and after the 2-week intervention period to track behavioral changes and exercise-induced neuroplasticity, respectively (see Fig. 1). Training of the intervention group lasted 2 weeks and comprised a total of 7 supervised training sessions, whereas the control group continued with their habitual activities (life as usual) in parallel. Before the experiment started, all subjects underwent aerobic fitness assessment (cycle ergometry). Endurance test results were used to derive individually tailored intensity prescriptions for the intervention group and to compare baseline fitness between groups.

### Cardiovascular Fitness Assessment

Before starting the experiment, all participants performed a graded incremental exercise test (GXT) on a bicycle ergometer (Ergoline ergoselect 200, Bitz, Germany). We used the standard scheme of the World Health Organization (WHO; Hollmann et al. 2012) with an initial work intensity of 25 W and an increase of 25 W every 2 min (pedaling rate 60–70 per min). The GXT was terminated after completion of the stage during which a heart rate of 170 beats/min was reached. Heart rate was continuously monitored (Polar Elektro Oy H7, Kempele, Finland) and capillary whole blood samples were taken from the hyperemic earlobe 15–25 s before the end of each GXT stage.

Body weight-adjusted power output (physical working capacity, PWC) at fixed heart rates of 120 beats/min (PWC120) and 170 beats/min (PWC170) was determined by linear interpolation of the workload–heart rate pairs (Eston and Reilly 2001). Lactate concentrations were determined photometrically using a laboratory analyzer. Workload–lactate pairs were fitted with a third degree polynomial and two body weight-adjusted indices of cardiovascular fitness were calculated. These were the workload at a fixed lactate concentration of 3 mmol/L (P_3_) and the individual anaerobic threshold (IAT) determined with the “1.5 mmol method” as described elsewhere (Dickhuth et al. 1999). Body weight-adjusted P_3_ and IAT are both recognized as valid indicators of maximal lactate steady state and therefore cardiovascular fitness (Föhrenbach et al. 1987; Faude et al. 2009).

Note that we did not schedule a GXT post-test because we did not expect fitness gains exceeding a familiarization effect in a period as short as 2 weeks. Along these lines, research in sports medicine has shown that important endurance performance-related adaptations such as increased capillarization, increased left-ventricular volumes, or the transformation of fast-twitch-glycolytic (FTG) to fast-twitch-oxidative (FTO) muscle fibers typically take several months of training to occur (Shephard 2000).

### Cardiovascular Exercise Intervention

The training of the intervention group lasted 2 weeks and comprised a total of 7 supervised and individually tailored training sessions (rate of adherence = 100%). The primary aim of the training intervention was to repeatedly expose subjects to exercise-induced hyperlactatemia (Brooks 2018; Hashimoto et al. 2018; El Hayek et al. 2019) within the 2-week intervention period, but without evoking an undesired overtraining/overreaching state (Billat et al. 1999).

To this end, each training session started with continuous cycling at PWC120 for 5 min. This was immediately followed by a 3-min-phase with a gradual increase of exercise intensity in 6 steps of 30 s each, up to the individual’s 100% PWC170. This first intensity peak was followed by an another 4-min-phase at PWC120 and another 3-min-phase of stepwise increasing workload up to PWC170. Exercise ended with a cooling down phase at PWC120 for 4 min (overall session duration 19 min). For the last 4 training sessions, the duration of the 2 intensity peaks was increased to 4 min in each case, while the rest of the protocol remained unchanged (Lehmann et al. 2020). An increase of exercise intensity in the second week was chosen to avoid a habituation effect potentially resulting in a reduced neuroplastic response (Knaepen et al. 2010).

For each subject, we drew blood samples from the earlobe in the first training session of week 1 (19 min program) and the first training session of week 2 (21 min program), respectively. Sampling was performed 5 times during the respective training sessions. To assure that exercise-induced lactatemia took place, we first averaged the lactate concentrations of each training session separately before averaging the resulting values for both training sessions together. The resulting mean lactate value was subsequently normalized to the IAT (in terms of the absolute lactate value) of every subject and compared against µ_0_ = 100 by means of a one-sample *t*-test. Average training lactate values were significantly (mean difference = 39.76%, 95% CI [24.51, 55.01]) higher than the IAT, *t*(31) = 5.32, *P* < 0.001, *d* = 0.94. We conclude from this that the intervention was successful in straining the anaerobic lactic metabolism.

### Cognitive Assessment

#### Sustained Attention

To assess participants’ sustained attention and visual scanning speed-accuracy (Bates and Lemay 2004), we administered the German paper and pencil version of the revised “d2 Test of Attention” (d2-R; Brickenkamp et al. 2010). The d2-R consists of 14 test lines with 47 symbols on each line. Symbols are lowercase letters “d” or “p” marked with 1, 2, 3, or 4 small dashes above and/or below the letter. The test taker is asked to mark all occurrences of the letter “d” with 2 dashes as quickly and accurately as possible. All other characters are distractors that should be ignored. Symbols are processed from left to right, one line at a time. Every 20 s, the examiner instructs the test taker to proceed to the next line. We used concentration performance as a dependent variable, which is defined as the number of marked distractors (sum of errors of commission and errors of overlooking) subtracted from the total number of processed targets (Brickenkamp et al. 2010).

#### Processing Speed

Processing speed, an integral part of general intelligence (Vernon 1993), was measured by the “Zahlen-Verbindungs-Test” (ZVT; Oswald and Roth 1987). The ZVT is a trail-making test in which subjects are asked to connect the numbers 1–90 with a pen in ascending order as fast as they can. The next higher number is always located in an adjacent position. Four different forms of the test, each consisting of 90 items, are administered in a row. The total test score is calculated by dividing the total time taken to complete all forms by 4 (Oswald and Roth 1987).

#### Short-Term Memory

For assessment of short-term memory, we used the digit span subtest from the “Hamburg-Wechsler-Intelligenztest für Erwachsene” (HAWIE-R; Tewes 1991), which is the German equivalent of the revised “Wechsler Adult Intelligence Scale” (WAIS-R; Wechsler 1981). In this test, the examiner presents digit sequences at a rate of one per second, which must be recalled by the test taker in order (forward) or in reverse order (backward). Subjects had 2 trials per span length and continued with the next level after at least one successful trial. Testing was aborted when a participant consecutively failed two trials of the same span length. The total number of correctly remembered trials (forward and backward) was used as a dependent variable in the statistical analyses.

### MR Image Acquisition

MRI data were acquired on a 3 T MAGNETOM Prisma system (Siemens Healthcare) using a 32-channel head coil. We used the same protocol for each volunteer and each scanning session. Whenever possible, subjects were measured at approximately the same time of day during the study. The imaging protocol consisted of a series of MRI sequences, as outlined below. Subjects were asked to relax, to think of nothing in particular, and to move as little as possible. With respect to the functional image acquisitions, they were additionally instructed to stay awake and alert while keeping their eyes closed (Raichle et al. 2001). A pillow was placed surrounding the sides and the back of the head to minimize head motion.

Whole-brain diffusion-weighted images were acquired from 88 axial slices with a spatial resolution of 1.72 × 1.72 × 1.7 mm^3^ (no gap) with a twice-refocused spin echo echo-planar-imaging sequence (Reese et al. 2003): TE = 80 ms, TR = 11 000 ms, α = 90°, FOV = 220 × 220 mm^2^, matrix: 128 × 128, parallel imaging: GRAPPA acceleration factor 2 (Griswold et al. 2002), 60 diffusion-encoding gradient directions, *b*-value = 1000 s/mm^2^. Additionally, 7 datasets without diffusion weighting (*b* = 0 s/mm^2^) were acquired initially and interleaved after each block of 10 diffusion-weighted images as anatomical reference for off-line motion correction. The diffusion MRI sequence lasted ~15 min. A total of 46 participants provided usable diffusion data at baseline and post-test. Two participants had to be excluded from the analyses due to technological problems.

Resting state fMRI scans were acquired using T2*-weighted gradient-echo EPI (GE-EPI) with multiband acceleration, sensitive to BOLD contrast (Feinberg et al. 2010; Moeller et al. 2010). A total of 420 whole-brain volumes were acquired using the following parameters: axial acquisition orientation, phase encoding = A ≫ P, echo spacing = 0.67 ms, voxel size = 2.3 mm isotropic, FOV = 202 × 202 mm^2^, matrix = 88 × 88, 64 slices with 2.3 mm thickness, TE = 30 ms, TR = 1400 ms, α = 69°, partial Fourier factor = 7/8, multiband acceleration factor = 4, acquisition bandwidth = 1775 Hz/Px, interleaved slice order. The total acquisition time for rs-fMRI was ~10 min.

T1-weighted anatomical images to investigate gray matter volume (GMV) and pulsed arterial spin labeling data to investigate cerebral blood flow (CBF) were also acquired and processed. Since analyses based on these data did not yield significant results or statistical trends in the present study, they are not discussed further (see Lehmann et al. 2020, for details).

### Processing of MR Images

All MRI modalities were preprocessed as extensively described in a previously published paper (Lehmann et al. 2020). In the following, we therefore focus on a brief description of the applied diffusion and rs-fMRI preprocessing pipelines.

Diffusion imaging datasets were processed using the FMRIB Software Library v5.0.9 (https://fsl.fmrib.ox.ac.uk/fsl/fslwiki/FSL; Smith et al. 2004). We started by checking for visual artifacts, followed by skull stripping and motion correction (Jenkinson et al. 2002; Leemans and Jones 2009). The diffusion indices fractional anisotropy (FA), mean diffusivity (MD), and radial diffusivity (λ_⊥_) were computed from the eigenvalues of the diffusion tensor with the respective formulas (Pierpaoli and Basser 1996). Subsequent steps followed the tract-based spatial statistics (TBSS) approach (Smith et al. 2006), including creation of a subject-specific template in midspace (Engvig et al. 2012; Madhyastha et al. 2014), normalization of the subject-specific templates to MNI space via the most representative template of the sample, and averaging/skeletonization. Finally, all midspace registered FA, MD, and λ_⊥_ maps were projected onto the skeleton using the warp fields created previously.

The rs-fMRI data were preprocessed using the toolbox fMRIPprep 1.1.3 (Esteban et al. 2019) including corrections for motion, slice timing, and susceptibility distortions as well as intrasubject registration to the T1-weighted image and spatial normalization. Further denoising included an automatic removal of motion artifacts using independent component analysis (ICA-AROMA; Pruim et al. 2015), linear detrending, high-pass filtering, and corrections for the global signal in the white matter and the cerebrospinal fluid. Note that all nuisance regressors were orthogonalized with respect to the ICA-AROMA noise components before the denoising was performed (Esteban et al. 2019; Lindquist et al. 2019).

For the subsequent analysis of resting-state functional connectivity, we quantified two centrality measures based on a graph theoretical approach. Degree centrality (DC) summarizes the connection strengths of each node to all other nodes in the network (Wink et al. 2012; van Duinkerken et al. 2017). In contrast, eigenvector centrality (EC) reflects the relative importance of a node to the network as a whole, such that high centrality values are assigned to nodes which are correlated with many other nodes that themselves are “central” (Lohmann et al. 2010). DC and EC for each subject and time point were computed at the voxel-level within a study-specific gray matter mask using the toolboxes fastECM (Wink et al. 2012; van Duinkerken et al. 2017) and LIPSIA v3.0 (Lohmann et al. 2010; Lohmann et al. 2018), respectively, and therefore did not require any a priori parcellation.

#### Seed-Based Correlation Analysis and Probabilistic Diffusion Tractography

In order to unfold the spatial topography of functional connectivity (centrality) results, seed-based correlation analysis (SBCA) was performed on preprocessed rs-fMRI datasets using FSL’s dual-regression tool (Nickerson et al. 2017). Initially, clusters that showed significant FWE-corrected nonparametric combination (NPC) effects (see Nonparametric combination) were used as a binary mask to extract the mean BOLD timeseries for each subject’s baseline measurement. To reconstruct the network topography, the extracted timeseries were regressed on the same 4D dataset to get a subject-specific whole-brain map containing standardized regression coefficients (β) and a correlation map with standardized (*z*-transformed) values. Afterwards, the *z*-transformed correlation maps were averaged across subjects and parcellated into distinct functional–anatomical clusters. To this end, a binary threshold of *z* > 2 was applied, for example, only voxels exceeding the within-mask mean correlation value by at least 2 standard deviations (SDs) were allowed to form clusters. This procedure was chosen because a nonself-referential task (task-based fMRI) to contrast with the resting-state activity was not at our disposal (Raichle et al. 2001; Raichle 2015).

The distinct functional–anatomical clusters constituting the resting-state network were then used as seed masks for probabilistic diffusion tractography (PDT) to reconstruct the underlying structural network. For this purpose, the BEDPOSTx (Behrens et al. 2003) and PROBTRACKx (Behrens et al. 2007) tools as implemented in FSL were used. BEDPOSTx started with the estimation of a probability distribution function of fiber direction in each voxel, before a two-fiber model was fitted to the diffusion data at each voxel. All nodes (clusters) constituting the SBCA-derived network were registered into each subject’s native diffusion space and used as seed masks for the fiber tracking. One thousand streamlines were randomly seeded from each voxel belonging to a seed mask using modified Euler streamlining and default settings of PROBTRACKx (curvature threshold = 0.2, step length = 0.5 mm, maximum steps = 2000, fiber volume threshold = 0.01). Using PROBTRACKx’ network mode option, only streamlines originating in a voxel within one seed mask and passing through a voxel in another seed mask were retained. This provides an image of each subject in MNI152 space in which each brain voxel’s value represents its visitation counts (i.e., the number of successful streamlines passing through that voxel). To avoid spurious connections, individual images were thresholded and binarized at 1/1000 of the total number of successful streamline visitations (waytotal) (Brown et al. 2015). In the last step, the individual proportion images were summed across subjects and thresholded to display only paths that were present in a minimum of one-third of the subjects (Song et al. 2012).

### Statistical Analysis

#### Statistical Analysis of Between-Group Differences at Baseline

Between-group comparisons at baseline were conducted dependent on the level of measurement and whether assumptions of Welch’s *t*-test were met (Ruxton 2006; Delacre et al. 2017). Therefore, results are reported as chi square, Mann–Whitney, or *t*-statistics. If not otherwise stated, statistical tests of significance carried out throughout the manuscript were performed two-sided. Note that the statistical tests applied below are suited to deal with the unequal number of observations in each group (i.e., statistical imbalance).

#### Statistical Analysis of Cognitive Test Data

To determine whether the intervention had evoked a change in cognitive test performance, we chose unpaired Welch’s *t*-tests to analyze prepost gain scores of cognitive performance (in %) as dependent variables. Note that Welch’s *t*-test performs well in terms of Type I and Type II error rates even in case of unequal sample sizes and variances (Ruxton 2006; Delacre et al. 2017). In case of significant results or meaningful statistical trends, we followed-up these results by assessing whether potential confounders like age, biological sex, or pretest performance of the cognitive test in question were related to the gain score. If so, we next applied a statistical procedure to factor out these confounders. To achieve this, we used a robust generalization of the Johnson–Neyman (JN) technique (Johnson and Neyman 1936) as proposed by Wilcox (2017). Unlike analysis of covariance (ANCOVA), the robust JN-ANCOVA does not require homogeneity of regression slopes and allows both between-group and within-group heteroscedasticity (Wilcox 2017). Note that violations of the aforementioned statistical assumptions are normally recognized as the most important problems with ANCOVA, especially if combined with unbalanced statistical designs (Harwell 2003). The basic idea underlying the JN-procedure is to compare the estimated dependent variable scores of the groups at specific design points (i.e., covariate values) and then control the probability of one or more Type I errors using a critical value based on a K-variate Studentized maximum modulus distribution with infinite degrees of freedom (for a detailed account, see Wilcox 2017). We used a least-squares regression estimator, which provides adequate control over the Type I error probability (Wilcox 2017), along with resampling-based (*B* = 5000) estimation of the standard error (SE) of the predicted scores of the groups at the chosen design points. Regression lines were compared at the 25th and 75th percentile of the observed pretest cognitive test scores as well as at 8 points evenly spaced between these two values. Robust JN-ANCOVAs were calculated using the function ancJN as implemented in the WRS v0.36 package (Wilcox and Schönbrodt 2019) running in R v3.6.1 (R Development Core Team 2013).

#### Statistical Analysis of Exercise-Induced Plasticity

Whole-brain voxel-wise analyses on all imaging modalities were carried out using general linear models (GLM) and permutation-based nonparametric testing (FSL “randomise”; Winkler et al. 2014). To address the effect of the CE intervention on structural/functional brain changes, we created percentage change images (Δ) between baseline and postintervention for each imaging modality. The resulting change images were then analyzed separately using a GLM with age (Mills et al. 2016), sex (Ruigrok et al. 2014), and the baseline measurement of the respective imaging modality (Voss, Heo, et al. 2013) as covariates of no interest. Based on previous longitudinal neuroimaging studies, we modeled greater increases (directional *t*-contrasts) with respect to Δ_FA, Δ_EC, and Δ_DC in the intervention group compared with the control group. The opposite contrast weights were used for Δ_MD and Δ_λ_⊥_.

To accommodate unequal group variances due to the unbalanced statistical design (Behrens–Fisher problem), two permutation blocks reflecting the group assignment of the subjects were defined (Winkler et al. 2014). A total of 5000 within-group sign-flippings of the data were generated to build up the empirical null distribution from which statistical inference was performed (Aspin–Welch’s *v*-statistic; Aspin and Welch 1949; Winkler et al. 2014). Threshold-free cluster enhancement (TFCE; Smith and Nichols 2009) was used to enhance cluster-like structures in the statistical images without the need to define clusters beforehand in a binary manner. Cluster-based family-wise error (FWE) correction was applied to the statistical maps by using the distribution of the maximum statistic (Smith and Nichols 2009; Winkler et al. 2014). Voxels were considered significant at *P*-values of <0.05, FWE-corrected. Potentially meaningful statistical trends were assessed with a more liberal FWE-corrected threshold of *P* < 0.1. To localize the results in stereotactic space, we used the Harvard-Oxford cortical atlas (EC, DC) and the JHU white-matter tractography atlas (FA, MD, λ_⊥_) as implemented in FSL (Desikan et al. 2006; Hua et al. 2008).

#### Nonparametric Combination

In case of significant results emerging from the behavioral analysis of cognitive test data, we tested whether CE influences the changes in cognitive performance through functional and structural brain changes. To establish a pattern of results consistent with mediation, it needs to be shown that the causal variable “group” is correlated with the putative mediator “neuroplasticity” (“action theory”), and that the putative mediator is in turn correlated with the outcome “cognitive performance changes” (“conceptual theory”; MacKinnon et al. 2013).

To this end, whole-brain voxel-wise statistical analyses were carried out within a modified NPC framework using the Permutation Analysis of Linear Models v. alpha115 toolbox (PALM; Winkler et al. 2014; Winkler et al. 2016) running in a Matlab R2017B environment. In brief, NPC works by first analyzing the aforementioned univariate submodels separately (i.e., ANCOVA for “action theory,” and multiple regression for “conceptual theory”) using synchronized permutations (Winkler et al. 2016). With respect to the modalities Δ_FA, Δ_EC, and Δ_DC, we modeled the following directional *t*-contrasts: greater CE-induced structural/functional brain changes in the intervention compared with the control group (corrected for the influence of age and sex), and a positive correlation between structural/functional brain change and change in cognitive performance (corrected for the influence of age, sex, and group). The opposite contrast directions were used for Δ_MD and Δ_λ_⊥_. The analyses were conducted on residualized change scores of cognitive performance and brain measures (MacKinnon et al. 2013; Voss, Heo, et al. 2013), respectively, for example, the variance associated with the baseline was eliminated. According to the union–intersection principle (Pesarin and Salmaso 2010; Winkler et al. 2016), the resulting pieces of evidence were then aggregated using Fisher's (1932) combining function. This creates a joint statistic indicating whether the observed results are consistent with the global (mediation) hypothesis. Note that the “global” null hypothesis of the NPC is that all null hypotheses for the partial tests are true whereas the alternative hypothesis is that any is false (Winkler et al. 2016). The joint statistic is significant if an aggregate of the partial tests is significant (Pesarin and Salmaso 2010; Winkler et al. 2016). This is also compatible with current thinking in statistical mediation analysis (Preacher and Hayes 2008).

As with CE-induced plasticity analysis (previous section), we ran the NPC with 5000 within-group sign-flippings. Clusters were formed using TFCE (Smith and Nichols 2009) and tested for significance at *P* < 0.05 (cluster-based FWE-correction).

#### Mediation Analysis

If the union–intersection test (UIT) (NPC) across directional *t*-contrasts yields the anticipated pattern of results, we directly tested the hypothesis that group assignment is related to brain plasticity, which in turn predicts cognitive performance changes. To this end, we used a regression-based approach to mediation (Preacher and Hayes 2004). The approach evaluates the decline in the strength of the relationship between predictor (group) and outcome (changes in cognitive performance) when controlling for the influence of the mediating variables (brain plasticity).

Initially, significant clusters emerging from the whole-brain analyses were used as a mask for averaging and extracting voxel values of residualized change of the respective modality for each subject. To determine whether neuroplasticity mediates the relationship between the intervention (binary-coded as: control = 0 and intervention = 1) and changes in cognitive performance, we calculated parallel mediation models with bootstrap confidence interval (CI) estimation as implemented in the PROCESS v3.4.1 macro (Preacher and Hayes 2004) running under an IBM SPSS v24 (Armonk, NY) environment. Resampling-based estimation of the mediated effect imposes no distributional assumptions (Preacher and Hayes 2008) and has shown to be applicable even in the case of small samples (*n* ≈ 25; Shrout and Bolger 2002; Preacher and Hayes 2004). To keep variation due to the random resampling process to an absolute minimum, 50 000 bootstrap samples were drawn using the percentile method. A heteroscedasticity-consistent SE and covariance matrix estimator was used (White 1980). From each of the bootstrap samples the indirect effect was computed and the sampling distribution was empirically generated. With the distribution, 95% CI (percentile 95% CI) were determined. A significant mediating effect is assumed if the percentile 95% CI of an indirect effect does not contain zero (Preacher and Hayes 2008). Note that statistical analyses were conducted on residualized change scores of the mediators and the criterion (MacKinnon et al. 2013). Age and sex were added to all models as covariates of no interest.

## Results

### Baseline Comparisons

No pre-existing between-group differences regarding demographic, anthropometric, or aerobic fitness data were detected (all *P*’s ≥ 0.24; Table 1). Subjects in the intervention group exercised with lactate values that were on average 40% higher compared with their IAT, such that the intervention was successful in straining the anaerobic-lactic energy system (see Methods).

### Exercise Improves Visual Processing, but not Information Processing Speed and Short-Term Memory

**Figure 2:**
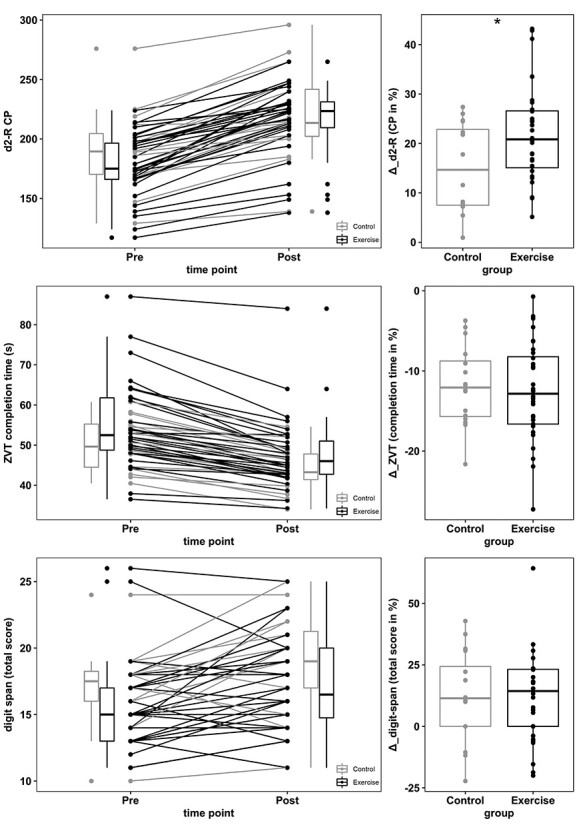
Effect of the CE intervention on sustained attention (d2-R concentration performance [CP]; Brickenkamp et al. 2010), processing speed (ZVT; Oswald and Roth 1987) and short-term memory (digit span; Wechsler 1981; Tewes 1991). Pre and post values for all cognitive domains are shown on the left-hand side, percentage gain scores on the right-hand side. Asterisk indicates statistical significance at *P* < 0.05 (Welch’s test on between-group difference in prepost change scores).

The next set of analyses investigated the effect of CE on cognitive performance using Welch-tests (Delacre et al. 2017) with change scores as dependent variables. There was a significant effect of the intervention on d2-R performance changes, *t*(32.82) = −2.35, *P* = 0.02, mean difference = 6.62%, 95% CI = [−12.35, −0.89], *d* = 0.71 (see Fig. 2). Analyses did not reveal significant between-group differences regarding ZVT, *t*(35.77) = 0.42, *P* = 0.67, mean difference = 0.69%, 95% CI = [−2.62, 4.01], *d* = 0.13, and digit span, *t*(28.96) = 0.15, *P* = 0.88, mean difference = 0.85%, 95% CI = [−10.50, 12.20], *d* = 0.05, respectively (Fig. 2). Because the Welch-tests did not show statistical trends for ZVT and digit span, subsequent analyses focused exclusively on d2-R performance changes.

With regard to confounding variables, we found that baseline d2-R performance significantly predicted the percentage gain score, *r* = −0.33, *P* = 0.02, while age, *r* = 0.06, *P* = 0.69, and sex, *t*(36.65) = −0.44, *P* = 0.66, did not. Note, however, that groups did not differ regarding d2-R baseline performance, *t*(24.58) = 1.35, *P* = 0.19, mean difference = 13.09, 95% CI = [−6.93, 33.12]. In order to correct for the influence of baseline performance, a robust JN-ANCOVA was applied (Johnson and Neyman 1936; Wilcox 2017). Estimated group means (Table 2) generally indicated that between-group differences decreased with higher d2-R pretest scores, and increased when lower. Significant differences favoring the group that received intervention were obtained for pretest d2-R values between 170 and 185, and marginal group differences (*P* < 0.1) were present in a range from 188 to 195. For d2-R pretest scores of ≥199, estimated between-group differences were no longer significant.

**Table 2:** Between-group comparison of d2-R performance changes at 10 design points between the 25th and 75th percentile of pretest d2-R performance based on JN ANCOVA (Johnson and Neyman 1936; Wilcox 2017)

CP pretest score	Control group (estimated value)	Exercise group (estimated value)	Test statistic	SE	*P*-value
167	16.42	23.12	−1.95	3.43	0.051
170	16.24	22.68	−2.00	3.22	0.046
174	16.05	22.24	−2.03	3.05	0.042
177	15.86	21.80	−2.05	2.90	0.041
181	15.67	21.36	−2.04	2.80	0.042
185	15.48	20.91	−1.99	2.73	0.047
188	15.29	20.47	−1.91	2.72	0.056
192	15.10	20.03	−1.80	2.75	0.073
195	14.91	19.59	−1.66	2.83	0.097
199	14.72	19.15	−1.51	2.95	0.132

### Exercise Induces Changes in White-Matter Architecture

**Figure 3:**
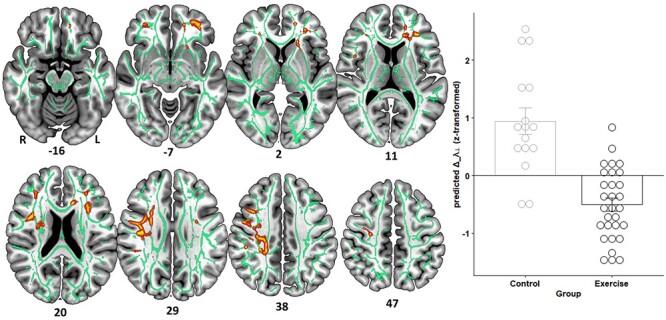
Radial diffusivity changes (Δ_λ_⊥_) induced by the intervention differ between groups (corrected for the variance associated with baseline λ_⊥_, age, and sex). Clusters are displayed at *P* < 0.05, FWE-corrected (TFCE) and thickened with the “tbss_fill” script for better visualization. Refer to Table 3 for anatomical description and MNI coordinates of significant clusters. On the right-hand side, the underlying data (within-cluster average) are presented as predicted values (dots) and associated estimated marginal group means (EMM) in SD units. Error bars represent 1 ± SE of the EMM. Note that *z*-scores < 0 indicate subjects whose Δ_λ_⊥_ decreased more than could be linearly predicted from the covariates, and reverse for *z*-scores > 0.

**Figure 4:**
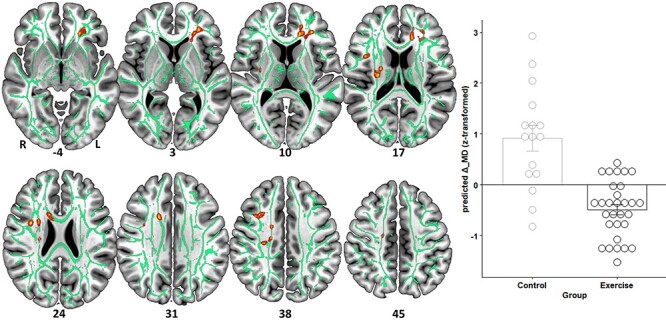
Mean diffusivity changes (Δ_MD) induced by the intervention differ between groups (corrected for the variance associated with baseline MD, age, and sex). Clusters are displayed at *P* < 0.05, FWE-corrected (TFCE) and thickened with the “tbss_fill” script for better visualization. Refer to Table 3 for anatomical description and MNI coordinates of significant clusters. On the right-hand side, the underlying data (within-cluster average) are presented as predicted values (dots) and associated EMM in SD units. Error bars represent 1 ± SE of the EMM. Note that *z*-scores < 0 indicate subjects whose Δ_MD decreased more than could be linearly predicted from the covariates, and reverse for *z*-scores > 0.

To determine the impact of CE on neuroplasticity, we tested for significant interactions between group and time with respect to structural and functional brain changes (corrected for the variance associated with baseline variations in brain structure/function, age, and sex). We found that the intervention was effective at inducing plasticity mainly in fronto-temporal white matter fiber tracts (Figs 3 and 4, Table 3). That is, λ_⊥_ and MD decreased more in the intervention compared with the control group (*p*FWE < 0.05). We also found trend-level results (*p*FWE < 0.1) for exercise-induced FA (Supplementary Fig. 1 and Supplementary Table 1) and DC increases (Supplementary Fig. 2 and Supplementary Table 1) compared with the life-as-usual control condition.

**Table 3:** Peak voxel coordinates and localization of significant clusters emerging from the voxel-based TBSS analyses (Figs 3 and 4)

Cluster index	Cluster extent	Maximum *P*-value	Peak voxel (MNI152)			Most prominent structures in clusters (Hua et al. 2008)
			*X*	*Y*	*Z*	
**Radial diffusivity (Δ_λ**_**⊥**_ **)**						
17	2511	0.036	35	3	26	Bilateral Superior Longitudinal Fasciculus, Bilateral Inferior Fronto-Occipital Fasciculus, Forceps Minor, Bilateral Anterior Thalamic Radiation, Bilateral Uncinate Fasciculus, Right Corticospinal Tract, Left Cingulum
16	1294	0.046	−25	35	6	
15	262	0.047	−27	52	−9	
14	141	0.048	32	36	20	
13	59	0.05	35	48	−9	
12	51	0.049	46	−31	38	
11	47	0.05	−43	41	−5	
10	35	0.05	27	37	0	
9	29	0.05	32	37	4	
8	22	0.05	31	17	24	
7	15	0.05	33	37	−3	
6	14	0.05	22	33	−5	
5	10	0.05	41	39	−2	
4	7	0.05	−17	45	16	
3	3	0.05	36	51	8	
2	3	0.05	36	47	−5	
1	1	0.05	43	39	−2	
**Mean Diffusivity (Δ_MD)**						
11	628	0.046	−25	36	−3	Bilateral Superior Longitudinal Fasciculus, Left Inferior Fronto-Occipital Fasciculus, Forceps Minor, Bilateral Anterior Thalamic Radiation, Left Uncinate Fasciculus, Right Corticospinal Tract, Left Cingulum
10	403	0.047	37	4	23	
9	147	0.047	15	13	26	
8	78	0.049	36	12	38	
7	59	0.049	25	−20	34	
6	50	0.049	19	−19	40	
5	38	0.049	32	−10	10	
4	18	0.049	−22	21	0	
3	16	0.05	30	−18	14	
2	16	0.049	17	−2	37	
1	7	0.05	2	17	17	

### Exercise-Induced Functional Connectivity Changes Mediate the Effect of Exercise on Visual Attentional Processing

**Figure 5:**
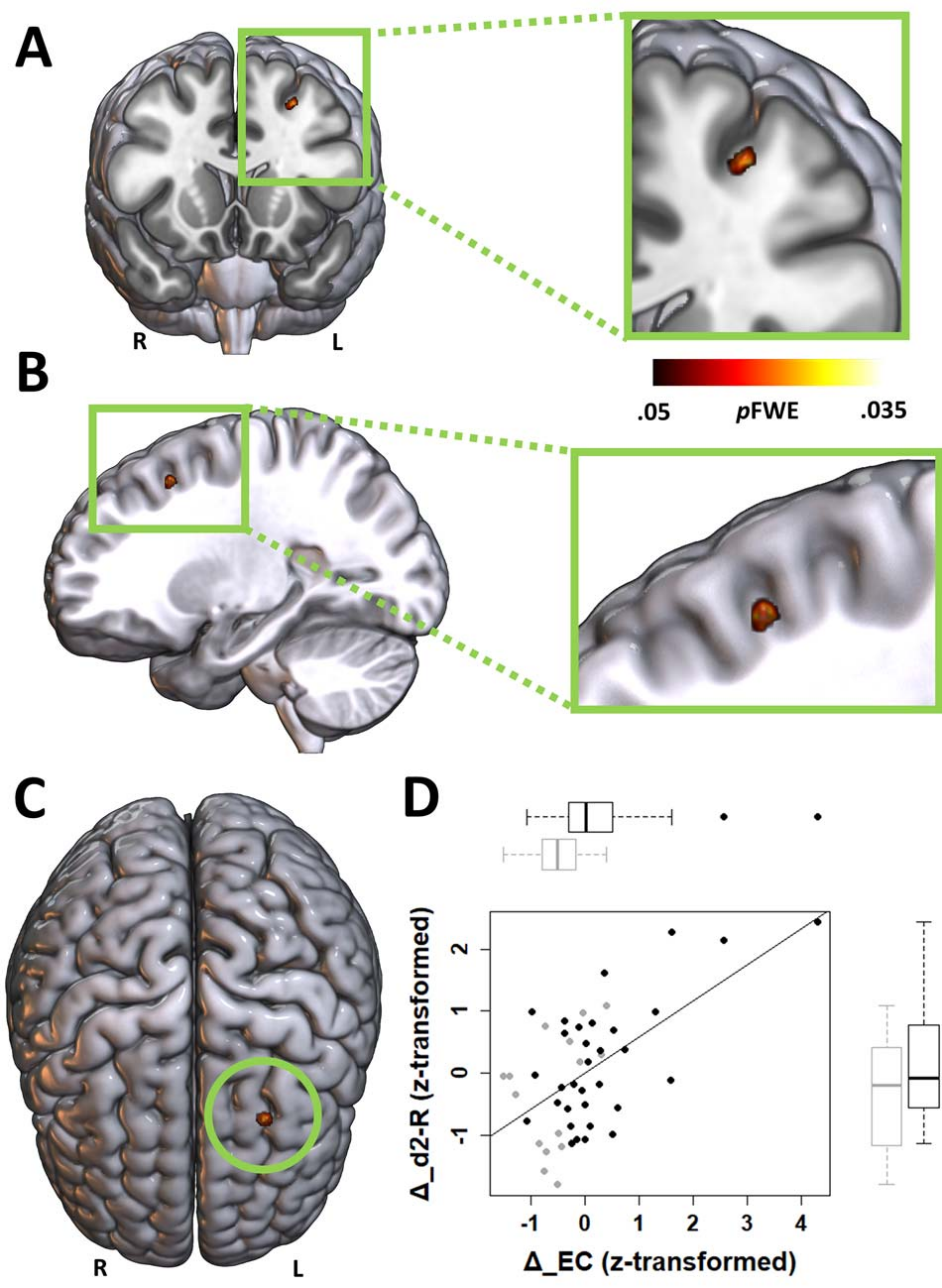
EC changes (Δ_EC) covary with both treatment (exercise vs. control) and the dependent variable concentration performance change (Δ_d2-R). *A*–*C*, Coronal, sagittal and superior view of the results from the UITs on baseline-adjusted (residualized) Δ_EC maps based on the NPC methodology. The significant cluster depicts voxels in which UITs revealed evidence for the presence of both a between-group difference with respect to exercise-induced residualized Δ_EC (corrected for the variance associated with age and sex), and a correlation between exercise-induced residualized Δ_EC and baseline-adjusted (residualized) Δ_d2-R (corrected for age, sex, and group). Color bar indicates FWE-corrected *P*-values. *D*, Partial regression scatterplot with line of best fit shows the relation between residualized Δ_EC (within-cluster average) and residualized Δ_d2-R in SD units, corrected for the influence of age and sex. Adjacent boxplots visualize between-group differences in Δ_EC and Δ_d2-R, respectively. Note that *z*-scores < 0 indicate subjects whose change scores decreased more than could be linearly predicted from the covariates, and reverse for *z*-scores > 0.

To evaluate whether structural and functional changes of the brain underlie the influence of CE on sustained attention, we continued our analyses by addressing the mediation hypothesis outlined previously. With respect to EC changes (Δ_EC), NPC analysis revealed a cluster at the anterior edge of the premotor cortex in the middle frontal gyrus (cluster extent *k* = 17, *p*FWE = 0.034, peak-voxel MNI coordinates: *x* = −28.5, *y* = 17.5, *z* = 47.5; Fig. 5). The pattern of results for the cluster is consistent with the global mediation hypothesis, for example, Δ_EC was higher in the exercise group compared with the control group, and there was a group-independent positive relationship between Δ_EC and changes in attention performance (Fig. 5*D*). Whole-brain NPC analyses of the other modalities did not yield any significant results.

**Figure 6:**
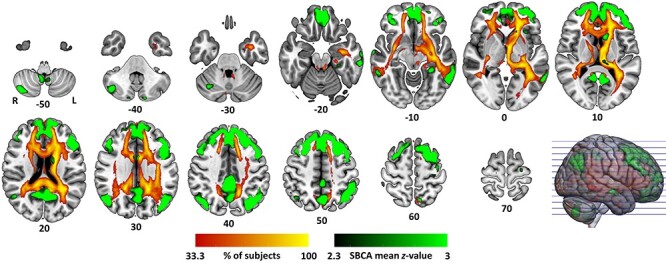
Reconstruction of the functional (green coloring) and structural networks (red–yellow) in which the significant EC-cluster from NPC (Fig. 5) is embedded in. Structural connectivity color bar refers to the percentage of subjects in whom the seeded streamlines passed through a given voxel.

As a next step, we used each subject’s averaged within-cluster BOLD timeseries (baseline scan) as a temporal regressor for an SBCA. The resulting BOLD coupling pattern allowed us to identify in which cortical network(s) the cluster potentially participates. Group-averaged seed-to-voxel connectivity maps (Fig. 6) suggest functional coupling of the EC-cluster especially with the ventromedial prefrontal cortex, the rostral anterior cingulate cortex, the posterior cingulate cortex, the rostral ventral and caudal ventral prefrontal cortex, the caudal inferior parietal lobule, the midsection of the inferior parietal lobule, and the precuneus. According to Sallet et al. (2013), this network topography is characteristic for a part of Brodmann area (BA) 8, which resembles area 8b in macaques (termed “cluster 10,” in the Sallet et al. paper).

**Figure 7:**
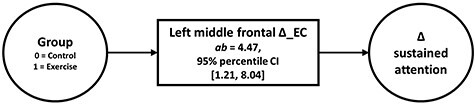
Exercise-induced neuroplasticity conveys the effect of treatment on changes in attention performance. The mediator model shows the relationship between allocation to treatment (Group) and baseline-adjusted (residualized) attention changes, transmitted via residualized Δ_EC and corrected for the influence of age and sex. CIs not including zero indicate significant indirect effects.

So far, the NPC results indicate that CE-induced functional changes in the left middle frontal gyrus could plausibly be a mechanism driving the CE–attention relationship. To directly test the presence of an indirect effect of group assignment (intervention vs. control) on attention performance changes via functional plasticity, residualized Δ_EC values within the significant cluster were extracted and used as an intervening variable in a statistical mediation analysis. The analysis revealed a significant indirect effect, indicating that EC changes mediated between group and attention changes (*ab* = 4.47, 95% percentile CI [1.21, 8.04], bootstrapped SE = 1.72; Fig. 7). Expressed as an effect size (partially standardized indirect effect), the intervention group’s change in attention performance was 0.48 SDs (95% percentile CI [0.15, 0.79], bootstrapped SE = 0.16) higher than the control group’s change as the result of EC changes.

To evaluate the robustness of our results, the analysis was repeated using a mediation procedure proposed by Zu and Yuan (2010) as implemented in R’s WRS v0.36 package (Wilcox and Schönbrodt 2019). This method downweights extreme cases using a Huber-type M-estimator in conjunction with a percentile bootstrap method. If the Huber-type weight is applied to control 5% of cases (*κ* = 0.05, as originally proposed by Zu and Yuan 2010), the percentile CI based on 50 000 bootstrap samples indicated that the indirect effect (*ab* = 3.23) was still entirely above zero (95% percentile CI [0.79, 6.15]). Even with a more conservative control of 20% of cases (*κ* = 0.2), the result remains significant (*ab* = 2.56, 95% percentile CI [0.55, 5.00]). Note that the Zu and Yuan (2010) method as implemented in WRS does not allow the inclusion of covariates, such that it was not possible to consider the influence of age and sex in this analysis.

### Relationship Between Structural and Functional Connectivity Changes

Potential inter-relationships were then assessed between longitudinal changes in structural and functional connectivity. Specifically, an exploratory cross-modal correlation analysis was used to analyze whether the clusters emerging from the previously conducted analyses are part of a common brain network.

To this end, we used PDT to reconstruct the anatomical links (edges) interconnecting the nodes of the resting-state network associated with the EC-cluster (Fig. 6). We identified voxels in which the reconstructed structural network intersected with significant clusters from the group-by-time interaction TBSS analysis (Exercise Induces Changes in White-Matter Architecture section). Overlapping voxels were mainly present in fiber tracts of the left hemisphere (inferior fronto-occipital fasciculus, anterior thalamic radiation, uncinate fasciculus, cingulum) but also in the forceps minor and the right superior longitudinal fasciculus (Supplementary Fig. 3).

The intersection masks were used to extract residualized Δ_λ_⊥_ and Δ_MD, which were then tested for correlation with left middle frontal EC changes (residualized Δ_EC). To reduce the impact of outliers, correlations were determined using Spearman’s-Rho. The results revealed a weak but significant correlation between Δ_λ_⊥_ and Δ_EC (*r_s_* = −0.30, *P* = 0.04). Likewise, a marginal significant correlation between Δ_MD and Δ_EC was found (*r_s_* = −0.27, *P* = 0.07).

## Discussion

The role of CE for brain health and cognition has been a popular area of research in recent years. At present, however, it appears that the question of how CE is related to cognitive functions in early adulthood is a blind spot of research (Erickson et al. 2019; Ludyga et al. 2020). This is particularly true for the identification of brain mechanisms mediating between CE and cognition in this age group (Stillman et al. 2016). For example, although previous cross-sectional studies with young adults suggest that aerobic fitness is associated with intrinsic connectivity of cognition-related brain networks (Raichlen et al. 2016; Talukdar et al. 2018), it remains unclear whether these changes are inherited or caused by CE, and whether they translate into improved cognitive performance. This longitudinal controlled trial adds to the scarce literature by demonstrating that a short duration CE intervention (2 weeks) is effective for augmenting sustained attention. Further, we provide evidence that this effect is mediated by CE-induced intrinsic functional connectivity changes in the left middle frontal gyrus. We also observed CE-induced changes in white-matter microstructure, collectively suggesting that functional and structural connectivity are malleable by appropriate training stimuli in young healthy adults.

At a behavioral level, the finding that a short duration CE intervention enhances attention aligns with meta-analytic evidence across age groups (Smith et al. 2010). In this respect, it has been suggested that CE contributes to a more economical allocation of attentional resources, which is also manifested in neuroelectric indices like the P300 component of the event-related potential (Gómez-Pinilla and Hillman 2013; Kao et al. 2020). To the best of our knowledge, however, only three previous longitudinal controlled trials have addressed the CE–attention relationship in young, healthy adults (Stroth et al. 2009; Woost et al. 2018; Stern et al. 2019). Like in the present study, Stroth et al. (2009) used the d2-R test (Brickenkamp et al. 2010) before and after a 6-week intervention period, and the group-by-time interaction effect trended toward significance in favor of the exercise group (*P* = 0.08, *d* = 0.70, medium effect size). Conversely, Stern et al. (2019) who measured selective attention and Woost et al. (2018) who assessed alertness and covert shift of attention did not find a beneficial effect of CE. This may indicate that treatment effects depend at least in part on the specific domain of attention that is assessed (see also Smith et al. 2010 and Ludyga et al. 2020, for the task-specificity of CE effects), and that CE especially influences sustained attention. Note in this respect that current theoretical discussions in the psychological sciences challenge the notion that “attention” can or should be regarded as a unitary construct (cf., Hommel et al. 2019). It is especially questioned whether different behavioral phenomena usually captured by the umbrella term “attention” are implemented by similar neural mechanisms, or whether they rely on similar elementary cognitive subprocesses (Allport 1993; Fan et al. 2002; Blotenberg and Schmidt-Atzert 2019; Hommel et al. 2019). The most distinguishing feature of sustained attention tasks is the demand to continuously allocate processing resources over time (Ballard 1996; Schweizer 2005; Blotenberg and Schmidt-Atzert 2019), and more research is warranted to determine whether and, if so, why CE “specifically” targets this domain of attention. Between-study heterogeneity regarding the adopted CE training (e.g., in terms of duration, intensity, frequency) might be another relevant factor contributing to different results across studies (van Praag et al. 2014; Erickson et al. 2019).

The most remarkable result to emerge from our data is that increased interconnectedness of the left middle frontal gyrus in the resting-state statistically accounts for the between-group differences in sustained attention. Although the results of a previous well-powered cross-sectional study (*n* = 1206, *M*_age_ = 28.8y) pointed in a similar direction, by showing that structural connectivity covaried with aerobic fitness and an aggregate score of cognition (Opel et al. 2019), it cannot be fully ruled out that the mediation effect was merely a by-product of inherited, nonmodifiable factors and not related to CE. The same problem applies, of course, also to other cross-sectional studies indicating that functional and structural brain measures mediate between physical activity/fitness and cognitive performance (see Stillman et al. 2016, for an overview). On the contrary, most previous longitudinal neuroimaging studies investigating the CE–brain–cognition relationship did not test for statistical mediation (e.g., Voss et al. 2010; Erickson et al. 2011; Voss, Heo, et al. 2013). Note that CE-induced structural (Stern et al. 2019) and functional changes (Prehn et al. 2019) of the dorsal frontal cortex were also found in other studies with young adults but the behavioral relevance of these changes was not addressed.

Therefore, as far as we are aware, the present study provides the first experimental evidence in support of a causal path linking CE to the augmentation of cognition via functional brain plasticity in young adults. Because EC refers to the “hubness” of a voxel or brain region (Lohmann et al. 2010), our findings would seem to suggest that the relative importance of the left middle frontal gyrus within the brain increased in response to CE. A closer look at the correlation between the spontaneous BOLD fluctuations of the significant cluster and the rest of the brain revealed a functional network resembling the connectivity pattern of area 8b in macaques, corresponding to a subregion of BA8 in humans (Sallet et al. 2013). Strikingly, nonhuman primate studies and lesion studies in humans converge in demonstrating that BA8 and its homologs are causally involved in high level regulation of visual attention (Petrides 2015). Combining this with the fact that the d2-R is a neurocognitive test addressing continuous visual scanning accuracy and speed over time (Bates and Lemay 2004; Brickenkamp et al. 2010) substantiates a mediating role for BA8 between CE and sustained attention performance.

Besides the evidence supporting a causal path linking CE-induced functional connectivity changes to sustained attention, we also found that the intervention group showed decreased λ_⊥_ and MD compared with the control group, mainly in fronto-temporal fiber tracts. A trend for increased FA after 2 weeks of CE was also found. Of note, parts of these significant clusters spatially intersected with the structural network interconnecting the different “nodes” of the dorsal frontal resting-state network, and there was a weak correlation between the magnitude of structural and functional connectivity changes. This is in good agreement with the results of a recent large-sample cross-sectional study demonstrating associations between aerobic fitness and FA (Opel et al. 2019). Large-scale white matter remodeling in response to CE was also found in previous intervention studies in young- to middle-aged subjects, although these studies used mixed interventions consisting of aerobic and strength exercises (Mueller et al. 2015; Svatkova et al. 2015).

Generally, the results of the present study are consistent with the notion that CE increases the efficiency of neurotransmission, particularly in those networks which are involved in attention. Which cellular and molecular events might give rise to CE-induced changes in structural and intrinsic functional connectivity? One hypothesis is that the CE-induced increase in brain fueling with lactate is related to the changes in resting-state functional connectivity and sustained attention. In this respect, previous work has suggested that lactate produced from active muscles, as a function of exercise intensity, can be shuttled to several “consumer” organs, among them the brain (Brooks 2018; Hashimoto et al. 2018; Magistretti and Allaman 2018). A necessary precondition allowing lactate to enter the brain is that exercise intensity is high enough to raise arterial lactate above baseline levels in the circulation (“lactate threshold”; cf., Ide et al. 2000), which was definitely the case in the present study (see Cardiovascular Exercise Intervention section). Within the brain, lactate is known to be involved in important neuroplastic responses to CE, for example the secretion of neurotrophins (Morland et al. 2017; El Hayek et al. 2019). Along these lines, Prehn et al. (2019) have found that increased CE-induced frontal lobe resting-state functional connectivity was paralleled by increasing levels of brain-derived neurotrophic factor (BDNF) in the circulation. Of course, brain connectivity is an energy-demanding process, and energy delivery is related to the communication rate of cortical hubs (Tomasi et al. 2013). In this respect, lactate might play an important role due to its pivotal contribution to the brain’s energy metabolism (van Hall et al. 2009; Boumezbeur et al. 2010; Brooks 2018). In line with this notion, Hashimoto et al. (2018) have recently shown that executive functions were highly correlated with the brain’s lactate uptake after repeated bouts of high-intensity exercise. Of note, muscle-derived lactate entering the brain might also be involved in triggering CE-induced white matter plasticity. In this respect, previous research suggests that brain lactate contributes not only to axonal myelination (Gundersen et al. 2015), but also to astrocyte plasticity (Lundquist et al. 2019; Lundquist et al. 2020). With respect to the latter, it has been shown that experience-induced changes of astrocytic processes are related to changes in the diffusion-imaging signal (Blumenfeld-Katzir et al. 2011; Sagi et al. 2012), which is also plausible against the background that astrocytes occupy a large fraction of the white matter (Sampaio-Baptista and Johansen-Berg 2017). Importantly, all the aforementioned plasticity mechanisms seem to be mainly dependent on (repeated) exposure to high exercise intensities, therefore indicating that the mediating effect of CE on cognition does not necessarily coincide with aerobic fitness gains (Etnier et al. 2006; Young et al. 2015).

Unlike other research carried out in the area of CE and cognition (cf., Smith et al. 2010; Erickson et al. 2019; Ludyga et al. 2020), we did not find a significant effect of CE on processing speed and working memory. There are several possible explanations for the absence of such effects. To begin with, it should be kept in mind that the estimated effect sizes of CE on processing speed and working memory, based on meta-analyses, are modest (Smith et al. 2010; Ludyga et al. 2020). Only large-sample studies would be able to reliably detect effects of such small magnitude. With respect to working memory, aggregated study results suggest that CE effects are less consistent compared with other cognitive domains (Smith et al. 2010). Another possibility is that the assessment instruments used to objectify processing speed and working memory might play a role (Ludyga et al. 2020). For example, Smith et al. (2010) showed that studies using the trail-making test (TMT, Part A) or the digit span test as dependent variables were on aggregate not successful in demonstrating significant CE effects. Interestingly, however, the more difficult version of the TMT (Part B), representing a test of executive function (Arbuthnott and Frank 2000), appears to be more susceptible to enhancement by CE (Smith et al. 2010). Consistently, two recent intervention studies in young healthy adults using high-intensity interval training elicited significant effects on TMT-B performance (Eather et al. 2019; Mekari et al. 2020), thus converging with other work indicating that executive functions are highly malleable by CE in young adults (Stroth et al. 2010; Johann et al. 2016; Stern et al. 2019).

We are aware that our research may have some further limitations. We fully acknowledge that random assignment of all subjects to the experimental conditions was not possible (see Participants and Experimental Design, for an explanation). We also acknowledge that the consideration of an active control group (cf., Lindheimer et al. 2020) would have further strengthened the conclusions regarding CE’s effectiveness in enhancing sustained attention. We can also not ensure that the groups enrolled in the study were comparable with respect to potential confounders like intelligence and socio-economic status (Frith and Loprinzi 2017), albeit we are not aware of studies indicating that these factors influence an individual’s responsiveness to CE interventions. Although the sample size in our study was comparable with other CE–neuroimaging studies (van der Stouwe et al. 2018), it may have been under-powered to detect below-moderate effect sizes at the behavioral level. Of course, this issue might also apply to the absent CE-effects with respect to CBF and GMV, respectively. Regarding the former, we expected improved perfusion in response to CE (Maass et al. 2015; Stimpson et al. 2018), which we also found to be a mechanism mediating between CE and motor learning (Lehmann et al. 2020). Potentially, it might be that the mediation effect in Lehmann et al. (2020) was disproportionately driven by the correlation between perfusion changes and learning, such that a larger sample is required to detect this effect in terms of a significant (learning-independent) group-by-time interaction. Regarding GMV changes in young adults, equivocal results are apparent in the past literature (van der Stouwe et al. 2018), This applies even to the most widely studied structure in relation to exercise, namely the hippocampus. Some studies report an increase in hippocampal volume (Thomas et al. 2016), whereas others reveal no effects (Woost et al. 2018), or even volume decreases (Wagner et al. 2015), therefore highlighting that further research in young adults is warranted. Finally, although our results fulfill the statistical requirements of an indirect effect (MacKinnon et al. 2013), this must not be confused with the existence of a causal effect linking CE to sustained attention via dorsal frontal cortex centrality. Therefore, an interesting perspective of CE–cognition research would be to directly manipulate the assumed mechanisms of action, for example, by means of noninvasive brain stimulation, in order to establish causality.

## Conclusion

In this paper, we examined the effects of CE on cognition in young adults and the underlying mechanisms of this relationship, a largely neglected issue in the past literature. Our data suggest that a 2-week CE intervention can improve sustained attention, and that this beneficial effect is mediated by increased intrinsic connectivity (hubness) of the left middle frontal gyrus. Importantly, the connectional fingerprint of this hub indicates that the finding may be embedded in a resting-state network relevant for attentional processes. At the same time, CE also led to white matter remodeling mainly in frontotemporal fiber tracts, which might at the same time be involved in the functional reorganization of the attention network. Given that sustained attention is crucial for everyday activities like reading, driving, or listening to a talk, the findings presented herein might have promising practical implications.

## Data and Code Availability Statement

Data availability: The datasets generated during and/or analyzed during the current study will be available on request from the corresponding author (N.L.) without undue reservation.

Code availability: All previously unpublished computer code used to generate results that are reported in the paper will be available on request from the corresponding author (N.L.) without undue reservation.

## Notes

The authors thank Joshua Grant, PhD for proofreading the manuscript. *Conflicts of Interest:* The authors declare that they have no conflict of interest.

## Funding

Federal Institute of Sport Science (IIA1-070613/12-13, ZMVI1-070610/14-16). The funders had no role in study design, data collection and analysis, decision to publish, or preparation of the manuscript.

## Author’s Contributions

N.L.: Conceptualization, Methodology, Investigation, Formal analysis, Writing—original draft, Visualization, Project administration.

A.V.: Conceptualization, Methodology, Resources, Supervision.

M.T.: Conceptualization, Methodology, Resources, Writing—Review and Editing, Supervision, Project administration, Funding acquisition.

## Supplementary Material

03_suppl_material_tgaa075
