# Supplementary material for: Intrinsic Connectivity Changes Mediate the Beneficial Effect of Cardiovascular Exercise on Sustained Visual Attention
Source: Cereb Cortex Commun. 2020 Oct 9;1(1):tgaa075. doi: 10.1093/texcom/tgaa075 (PMC8152900; doi:10.1093/texcom/tgaa075)
Supplement: 03_suppl_material_tgaa075 [file 03_suppl_material_tgaa075.docx]

**Intrinsic Connectivity Changes Mediate the Beneficial Effect of Cardiovascular Exercise on Sustained Visual Attention**

Nico Lehmann, Arno Villringer & Marco Taubert

**Supplementary Material**


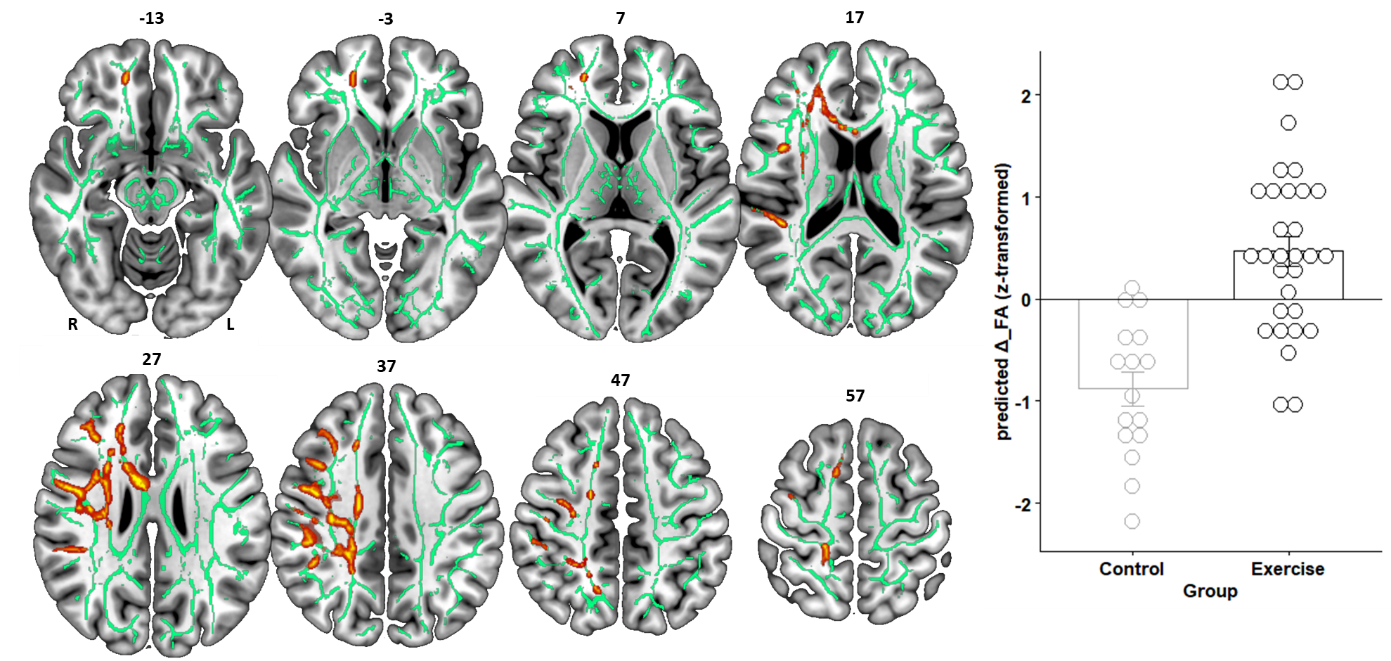


Supplementary Figure 1: Fractional anisotropy changes (Δ_FA) induced by the intervention differ between groups (corrected for the variance associated with baseline FA, age and sex). Clusters are displayed at p < 0.1, FWE-corrected (TFCE) and fattened with the “tbss_fill” script for the purpose of better visualization. Refer to Supplementary Table 1 for anatomical description and MNI coordinates of significant clusters. On the bottom side, the underlying data (within-cluster average) are presented as predicted values (dots) and associated estimated marginal group means (EMM) in standard deviation units. Error bars represent 1 ± standard error of the EMM. Note that z-scores < 0 indicate subjects whose Δ_FA decreased more than could be linearly predicted from the covariates, and vice versa.


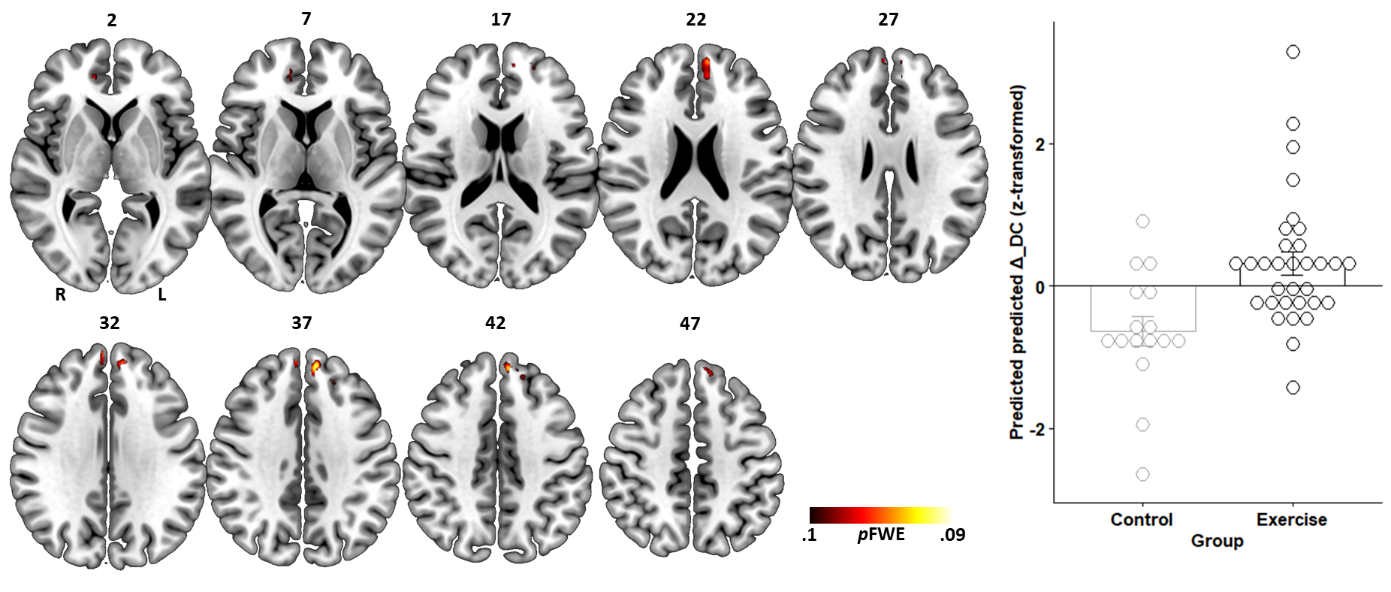


Supplementary Figure 2: Degree centrality changes (Δ_DC) induced by the intervention differ between groups (corrected for the variance associated with baseline DC, age and sex). Clusters are displayed at p < 0.1, FWE-corrected (TFCE) and fattened with the “tbss_fill” script for the purpose of better visualization. Refer to Supplementary Table 1 for anatomical description and MNI coordinates of significant clusters. On the bottom side, the underlying data (within-cluster average) are presented as predicted values (dots) and associated estimated marginal group means (EMM) in standard deviation units. Error bars represent 1 ± standard error of the EMM. Note that z-scores < 0 indicate subjects whose Δ_DC decreased more than could be linearly predicted from the covariates, and vice versa.

Supplementary Table 1: Peak voxel coordinates and localization of significant clusters emerging from the voxel-based fractional anisotropy (FA) and degree centrality (DC) analyses (Supplementary Figs. 1 and 2).

| Fractional Anisotropy (Δ_FA) | | | | | | |
| --- | --- | --- | --- | --- | --- | --- |
| Cluster Index | Cluster extent | Maximum *p*-value | Peak voxel (MNI152) | | | Most prominent structures in clusters (Hua et al. 2008) |
|  |  |  | X | Y | Z |  |
| 14 | 3712 | 0.066 | 46 | -1 | 25 | Right Superior Longitudinal Fasciculus, Forceps Minor, Right Corticospinal Tract, Right Anterior Thalamic Radiation, Right Cingulum, Right Inferior Fronto-Occipital Fasciculus, Right Uncinate Fasciculus, Right Inferior Longitudinal Fasciculus |
| 13 | 940 | 0.09 | 20 | 46 | 0 |  |
| 12 | 734 | 0.084 | 32 | 33 | 22 |  |
| 11 | 232 | 0.087 | 18 | -13 | 39 |  |
| 10 | 102 | 0.093 | 44 | 7 | 16 |  |
| 9 | 78 | 0.097 | 8 | 6 | 66 |  |
| 8 | 50 | 0.098 | 0 | 13 | 20 |  |
| 7 | 34 | 0.098 | 9 | 19 | 52 |  |
| 6 | 8 | 0.1 | 14 | 13 | 47 |  |
| 5 | 7 | 0.099 | 44 | -55 | 43 |  |
| 4 | 5 | 0.1 | 9 | 14 | 61 |  |
| 3 | 3 | 0.1 | 46 | -55 | 38 |  |
| 2 | 1 | 0.1 | 44 | -54 | 41 |  |
| 1 | 1 | 0.1 | 7 | 13 | 63 |  |
|  | | | | | | |
| Degree Centrality (Δ_DC) | | | | | | |
| Cluster Index | Cluster extent | Maximum *p*-value | Peak voxel (MNI152) | | | Most prominent structures in clusters (Desikan et al. 2006) |
|  |  |  | X | Y | Z |  |
| 5 | 238 | 0.091 | -8.5 | 47.5 | 39.5 | Frontal Pole, Superior Frontal Gyrus, Cingulate and Paracingulate Gyrus, Middle Frontal Gyrus, Frontal Medial Cortex |
| 4 | 61 | 0.093 | 3.5 | 57.5 | 29.5 |  |
| 3 | 30 | 0.097 | 13.5 | 45.5 | 3.5 |  |
| 2 | 8 | 0.099 | -22.5 | 53.5 | 17.5 |  |
| 1 | 6 | 0.098 | -12.5 | 65.5 | -0.5 |  |


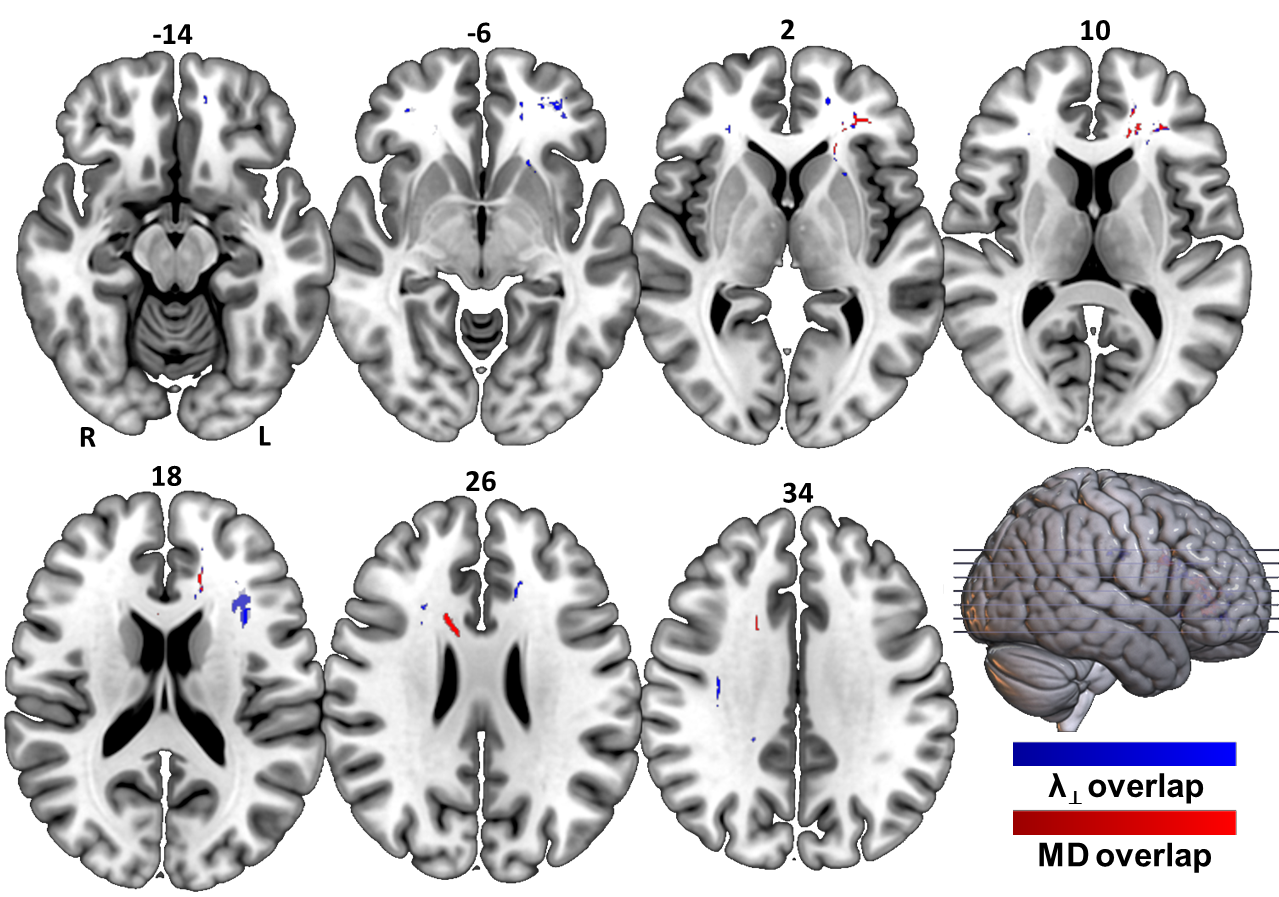


Supplementary Figure 3: Overview of voxels which were part of the white matter tracts interconnecting the resting-state network of the EC-cluster (main text, Figure 6) and which showed significant exercise-induced plasticity as well (group-by-time interaction TBSS analysis; main text, Figures 3 and 4).

References

Desikan RS, Ségonne F, Fischl B, Quinn BT, Dickerson BC, Blacker D, Buckner RL, Dale AM, Maguire RP, Hyman BT, et al. 2006. An automated labeling system for subdividing the human cerebral cortex on MRI scans into gyral based regions of interest. NeuroImage. 31(3):968–980.

Hua K, Zhang J, Wakana S, Jiang H, Li X, Reich DS, Calabresi PA, Pekar JJ, van Zijl PCM, Mori S. 2008. Tract probability maps in stereotaxic spaces: analyses of white matter anatomy and tract-specific quantification. NeuroImage. 39(1):336–347.
